# Inequality-Reducing Effects of Tobacco Tax Increase: Accounting for Behavioral Response of Low-, Middle-, and High-Income Households in Serbia

**DOI:** 10.3390/ijerph18189494

**Published:** 2021-09-09

**Authors:** Marko Vladisavljević, Jovan Zubović, Mihajlo Đukić, Olivera Jovanović

**Affiliations:** Institute of Economic Sciences, 11000 Belgrade, Serbia; jovan.zubovic@ien.bg.ac.rs (J.Z.); mihajlo.djukic@ien.bg.ac.rs (M.Đ.); olivera.jovanovic@ien.bg.ac.rs (O.J.)

**Keywords:** tobacco excises, price elasticity, tax progressivity, inequality, fiscal revenues

## Abstract

While previous research has indicated that increasing tobacco excises is a crucial instrument for lowering tobacco demand, this policy has been criticized for its alleged regressive impact on the poor. However, this critique does not take into account the behavioral response, i.e., decrease in consumption that occurs after excises and prices increase. In this paper, we examine the effect of cigarettes’ price increase on tobacco consumption, household expenditures, and tax burdens in three income groups and provide empirical arguments on the regressivity/progressivity effects of tobacco tax increase. Estimated elasticities indicate that all groups decrease their cigarettes demand with increasing prices, with demand decrease stronger for low- than for middle- and high-income households. Results further suggest that increasing tobacco excises (1) decreases tobacco expenditure of low-income households, which increases their productive consumption, such as on food, clothes, etc., and (2) redistributes the tobacco tax burden from low- to high-income households. Therefore, excise increase policies do not have an adverse effect on the position of the low-income households; on the contrary, they lower their cigarettes expenditure and their tax burden, while lower cigarettes consumption has an additional, positive effect on their health, which attenuates future inequalities.

## 1. Introduction

Although smoking prevalence in Serbia has been decreasing in recent years, it is still one of the highest in Europe. The share of daily smokers in Serbia in 2019 stood at 29.0% [1], while the comparable data for the EU-28 suggest a prevalence of 18.4% [2]. Other estimates indicate that, if both daily and occasional smokers are included, smoking prevalence in Serbia amounts to 37.4% [3]. High tobacco consumption represents a significant economic burden on the households, while, at the same time, the negative effects of tobacco consumption have long-lasting effects on health and well-being in general. Tobacco consumption has serious health consequences as approximately half of smokers die from tobacco-related diseases [4].

Numerous studies indicate that higher tobacco taxes and prices are one of the most important policies to reduce tobacco consumption [4], and high prevalence in Serbia is at least partially due to low prices of cigarettes, which, in 2018, stood at about €2 per pack, while the average price in the EU-28 was €4.8 [5]. Low prices of cigarettes are driven by low excises on tobacco products. In 2020, total excise stood at €1.47, while the minimum recommended excise in the EU countries stood at €1.8 [6]. At the same time, excise represents 58.7% of the weighted average price of cigarettes in Serbia, while the WHO framework convention on tobacco control, which Serbia signed in 2017, suggests that this share should be at least 70%. Additionally, the excise share has been decreasing since 2017, as the gradual increase of the specific tax (about 4% per year) is lower than the increase of the cigarettes’ prices in the same period (about 9% per year). Therefore, the excise increases served as an excuse to increase the net price of producers and, consequently, the profits of tobacco companies, while reducing the share of excises in retail price. Moreover, increases in taxes are not adjusted to increase in income, but rather only with inflation.

At the same time, Serbia has one of the highest at-the-risk-of-poverty rates in Europe, at 23.8%. While policies aiming at a more rapid tobacco excise and price increase have been criticized for their regressive impact on the poor, these arguments have been neglecting the changes in the consumption patterns that occur after the prices increase. The purpose of this paper was to empirically estimate the regressivity/progressivity effects of tobacco tax increase while accounting for the change in the consumption patterns that would occur after the tax increase. At the same time, we provided a test for the hypothesis that higher taxes on tobacco would have a disproportionally negative impact on the population with low income.

Previous research for Serbia [7] suggests a negative price elasticity in Serbia of −0.639, indicating that a 10% increase in prices can decrease the demand by about 6.4%. However, the evidence of the impact of price increase on different income groups for Serbia (or in the wider region of the Western Balkans) is non-existing. In this paper, we investigate how increases in cigarettes prices affect different income groups in Serbia, by using the Household Budget Survey (HBS) data for the years 2006–2017. We, (1) estimate the price elasticities separately for low-, middle-, and high-income households by accounting for both prevalence and intensity elasticity and combining the methodology from two-part [8] and Deaton’s demand [9] model and (2) provide a calculation of the effects of the tax increase on the consumption, expenditures, and tax burden for the three groups of households.

Results suggest that price elasticity is negative for all income groups, i.e., that when tobacco prices increase, all income groups decrease their demand. The elasticity is the highest for low-income households, estimated at −1.079 while for middle- and high-income households it is estimated at −0.632 and −0.260. These results, together with the subsequent analysis suggest that increasing tobacco taxes has several positive effects for the low-income households from the inequality perspective. Firstly, we showed that increasing excises would result in a decrease of overall tobacco expenditure for low-income households and that a part of the income that was used on cigarettes can now be used for other, more productive consumption purposes, such as on food, clothes, etc. This decreases the likelihood of so-called secondary poverty—the situation in which low-income households have enough resources but, due to inefficient use, they have the same level of productive consumption as poor households. Secondly, we showed that increasing tobacco excises would result in a tax burden shift towards high-income households, with low-income households paying a smaller share of total taxes. Finally, as low-income households decrease their demand the most when prices (or excises) increase, this means that increasing taxes on tobacco improves their health outcomes and lowers medical expenditures the most, which is important as they are most susceptible to tobacco-related diseases and mortality. 

Contributions of our paper are three-fold. Firstly, we present strong empirical evidence that disputes the argument that increasing tobacco prices would have adverse effects on low-income households in Serbia. On the contrary, increasing the prices, via lower consumption of cigarettes, lowers their cigarettes and medical expenditures, shifts the tax burden towards high-income households, and has positive effects on their health. Secondly, we provide first estimates of the price elasticities for the households from different income groups in Serbia and the wider region of the Western Balkans. Finally, we provide the first evidence on the separate effects of price increase on tobacco prevalence and intensity in Serbia.

This paper is structured as follows. After the introduction, in the literature review, we present the debate on the regressivity/progressivity of tobacco taxes and most recent research related to tobacco consumption, its relation to poverty, and its impact on different income groups. In the third section, we present the descriptive statistics and methodology used to estimate the price elasticities. In the fourth section, we present estimated price elasticities and the effects of price increase on consumption, expenditure, and tax burden by income groups. Section 5 presents conclusions and policy implications.

## 2. Literature Review: The Impact of Excise Taxes on Different Income Groups

The WHO review of the link between tobacco and poverty [10] suggests that low-income households have higher levels of tobacco prevalence and consumption. According to the review, this is valid for all continents, and particularly in the last two decades. From that perspective, tobacco excises have been viewed as regressive, as they put the highest burden on the low-income households [11]. Besides the higher levels of tobacco consumption, this argument can be taken as self-evident even if there were no consumption disparity: As the excises are approximately equal for all smokers regardless of their income levels (particularly in the case of specific excises), for the poor, excises represent a higher percentage of their income than for the rich. Hence, from this perspective, the taxes are regressive, and increasing excises and particularly specific excises will hurt the poor the most.

However, while this conclusion might be correct for the given taxes, this might not be true for the tax increase [11,12]. A vast body of research has indicated greater rsponsiveness of the poor on the price increase, i.e., higher tobacco price elasticity for low-income groups. In the UK, Townsend et al. [13] suggested that persons from the highest class do not change their demand when prices change, while, for the poorest class, the decrease in tobacco demand, as a consequence of the price increase, can be substantial. In the US, a person with low income has about 70 percent higher responsiveness to a price increase than a person with high income [14], while the less educated are also more price responsive than those with higher levels of education [15]. Therefore, for persons with high income, a tobacco excise increase will not result in a significant consumption decrease and, consequently, they will face a higher tax burden (in about the same percentage as taxes increase). On the other hand, for persons with low income, a tax increase will result in (about) a proportional decrease in demand, therefore, keeping the tax burden at approximately the same level. In other words, a tobacco excise increase (unlike a current tax) is a progressive policy: After accounting for the behavioral response, it will result in a tax increase for the rich (as a group), while, for the poor (as a group), they will remain the same [12]. Furthermore, in the long run, the poor will also have eventual gains in terms of better health and lower medical expenditures [11]. This is even more important as low-income groups are more susceptible to all tobacco-related illnesses, including lung, cardiovascular, and coronary disease, as well as tobacco-related mortality [10].

Similar findings have been suggested in recent research for middle- and low-income countries. Nargis et al. [16] used Deaton’s demand model [9] to calculate the price elasticity of cigarettes demand by income groups in Bangladesh. Estimated price elasticity was the highest for the low-income group, −0.75, while, for the other two income groups, price elasticities were significantly lower and amounted to −0.40 and −0.36, respectively. Mao et al. [17] also used Deaton’s model to estimate the price elasticity of demand for tobacco products in China and divide households into four income groups. Their results indicate that the two groups with the lowest income (the poor and low-income group) had the highest price elasticities and that the elasticity for the middle-income group was not significant, while for the high-income group, price elasticity was even positive (a price increase increases the demand for the product). Adioetomo et al. [18] suggested that, in Indonesia, the poorest smokers also had the highest response to changes in the price of tobacco products. The authors found that the price elasticity for the poorest was −0.67, for the middle-income group it was −0.33, and for the richest it was −0.31. Van Kinh et al. [19] and Choi [20] came to similar conclusions for Vietnam and South Korea. The price elasticities of demand for the poorest were −0.94 and −0.812, respectively.

Previous research for Serbia [7], which relied on Deaton’s demand model [9], estimated price elasticity in Serbia at −0.639, a level which is consistent with other research on the price elasticities in low- and middle-income countries [4]. This result indicates that, if the cigarettes’ prices increase by 10%, the overall demand would decrease by 6.4%. However, in this research, the authors did not calculate price elasticities by income groups or separate elasticities for smoking prevalence and intensity. In this research, we aimed to fill this gap, by estimating the price prevalence and intensity elasticity for three income groups and analyzing the implications of our results on the debate around the progressivity/regressivity of tobacco excise increase.

## 3. Materials and Methods

### 3.1. Data and Descriptive Statistics

To estimate the price elasticity of cigarette consumption in Serbia, Household Budget Survey (HBS) data from 2006 to 2017 was used. HBS is an annual, nationally representative survey, which provides detailed information on household consumption, as well as on individual characteristics of the household members. Additionally, the survey data contain information on the municipality and region in which the respondents live. In total, there were 62,053 households in the sample.

Table 1 presents the data on cigarette use available from HBS. Smoking prevalence, defined as the share of the households that reported positive cigarette expenditures, significantly decreased over the observed period: from 49.7% in 2006 to 34.2% in 2017, or by about 30%. Moreover, households decreased their smoking intensity: The average number of cigarettes smoked in the same period decreased from 39.1 to 27.2 packs per household per month, also by about 30%. Since only 1.7% of households in the sample reported expenditures on cut tobacco, this variable was not included in the analysis. Although there is a likely substitution effect between cigarettes and cut tobacco, the low number of households with positive cut tobacco consumption suggests that cut tobacco expenditures were not likely to impact the results. At the same time, however, household expenditures on cigarettes increased: The average household expenditure (among the households with positive expenditures) increased from 1988 RSD in 2006 to 3241 RSD in 2017 (expressed in 2006 values), or by about 63%. As the increase of household expenditure coincided with the lowering of the smoking intensity, this means that real cigarettes prices were growing faster than smoking intensity was declining.

HBS does not collect data on prices, so this analysis used a ratio of (real) household expenditure on cigarettes and the number of cigarettes smoked to calculate (real) unit values of cigarettes for each household. Average unit values of cigarettes reported by households within one municipality for each year was used as a proxy for cigarette price. Yearly trends of this variable are presented in the last column of Table 1. The average real price (proxy) of cigarettes increased from about 52 RSD in 2006 to about 118 RSD in 2017 (expressed in 2006 values), indicating that the real price of cigarettes increased by 2.3 times. Therefore, while the prices of cigarettes more than doubled in real terms over the observed 12 years, during the same period both smoking prevalence and smoking intensity decreased by about 30%. According to the official Statistics Office of the Republic of Serbia (SORS) data and our calculations, real tobacco Consumer Price Index (CPI) grew by 2.4 times, with similar trends by years, confirming the validity of the price measure that we used in our estimates.

#### Trends by Income Groups

**Figure 1:**
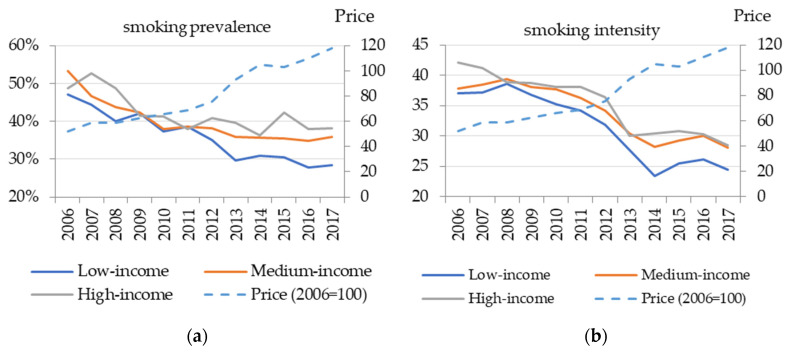
Smoking prevalence (**a**) and conditional (demand) intensity (**b**) trends by income group. Source: Authors’ calculation based on HBS data. Notes: Smoking prevalence is defined as the share of the households with positive tobacco consumption, while smoking intensity represents the number of cigarette packs a household with positive expenditures on cigarettes smoked per month. Cigarette prices are defined as municipality/year average cigarettes’ unit values (ratio between total expenditure and quantity) and are expressed in real terms (2006 = 100). Income groups are constructed based on total household consumption (a proxy for income) per capita.

Figure 1 (left panel) presents prevalence trends for the three income groups and compares them with the average prices for the period. Income groups are constructed based on total household consumption (a proxy for income) per capita for each year. The decrease in prevalence was the sharpest among low-income households, where the decrease was 18.6 percentage points (from 47 to 28.4%). The decrease was slightly lower for middle-income households—by 17.5 percentage points (from 53.4 to 35.9%), while the prevalence decrease of high-income households was below the average, at 10.7 percentage points (from 48.8 to 38.1%). Furthermore, in the period of the highest rise of prices (2011–2014), low-income households decreased their prevalence more than the two other income groups, indicating that low-income group prevalence trends might be more related to the price changes.

On the other hand, among the households with positive cigarette consumption, between 2006 and 2017, smoking intensity decreased on average by 11.9 packs per month. The decrease was above average in high-income households, by 13.7 packs (from 42.1 to 28.4 packs, or by about 32%), and in low-income households, by 12.6 packs (from 37 to 24.4 packs, or by about 34%). On the other hand, in middle-income households, the decrease was the lowest—9.7 packs (from 37.8 to 28.1 packs, or by about 26%). Like the prevalence trends, Figure 1 (right panel) indicates that, in the period of the highest rise of the prices (2011–2014), smoking intensity among low-income households decreased more than the two other income groups.

Therefore, in low-income households, the decrease in both smoking prevalence and intensity was higher than the national average. This resulted in unchanged real expenditures on cigarettes for low-income households (including both consuming and non-consuming households) in the period in which real prices of cigarettes more than doubled, while the budget share spent on cigarettes decreased by 0.4 percentage points (decrease from 3.3 to 2.9%). On the other hand, in the two other income groups, real expenditures increased by about 20%, which led to a slight increase in the total budget shares spent on cigarettes by 0.4 percentage points for middle-income households (from 3.0 to 3.4%) and by 0.8 percentage points for high-income households (from 2.4 to 3.2%).

This section offers descriptive evidence indicating that there is a negative correlation between the prices and tobacco prevalence and smoking intensity in the period between 2006 and 2017. However, this conclusion is based on the 12 observations and without considering other factors and correlates that might have impacted tobacco consumption. Therefore, in the next section, we discuss the regression analysis applied to analyze the effect of prices on smoking prevalence and intensity while controlling for the impact of other variables.

### 3.2. Econometric Methods for the Estimation of the Price Elasticity

#### 3.2.1. Theoretical Background and Econometric Model

The starting point of the estimation of price elasticity is the utility theory. Households seek to maximize the utility of their consumption, given their consumption preferences, prices, and budget restrictions. Therefore, the demand for each good is defined as a function of price, household budget, and other household characteristics. Since HBS is the household level data, we assumed that the household maximizes a single utility function. The demand for cigarettes is characterized by the high number of households that have no expenditures on smoking. From this perspective, households are facing two decisions. The household first decides whether to smoke or not smoke (extensive margin). If the household decides to smoke, they then decide how many cigarettes to smoke (intensive margin). This is also reflected in the variable describing cigarettes consumption, which is characterized by a mixed distribution that is partly discrete and partly continuous. The World Health Organization (WHO) estimates the worldwide proportion of smokers to be approximately 21% [21]. For non-smoking households, the variable describing the consumption takes a zero value, while the remaining outcomes are strictly positive.

The literature suggests that, when estimating determinants of this type of dependent variable, a two-part model should be used, in order to model the two decisions independently [8]. The first part of the model estimates cigarette prevalence, i.e., the probability of observing positive tobacco consumption (vs. no consumption), while the second part of the model deals with the intensity of cigarette consumption, where the (log) dependent variable is typically a linear function of independent variables. The latter part is conditional on positive cigarettes consumption; this part of the model is also called the conditional demand function.

The main variables that enter both prevalence and intensity models are price and income. These two variables provide the basis for the calculation of price and income elasticity of cigarette prevalence and the intensity of cigarette use. Aside from prices and income, the models include a set of covariates, consisting of household characteristics (share of men and adults in the household, maximum and mean level of education, and activity of the household members), region and settlement fixed effects, and variables representing institutional changes relevant to cigarette consumption. In general terms, the two-part model of price elasticity of cigarettes consumption can be written as:

(1) $$Y=P\left(y_{i}>0\right)=f\left(\beta_{1}p_{i}+\beta_{2}i_{i}+\Gamma^{\prime }X\right)$$

(2) $$E\left(y_{i}|y_{i}>0\right)=\alpha_{1}p_{i}+\alpha_{2}i_{i}+\Theta^{\prime }X$$

where $y_{i}$ is cigarette consumption of household *i*, and $p_{i}$ and $i_{i}$ are prices and total household consumption, respectively. *X* represents the vector of other covariates used in the analysis. Coefficients $\alpha_{1}$, $\alpha_{2}$, and Θ in the second part of the model represent marginal effects and are easily translatable into elasticities. As the first part of the model is typically estimated by a parametric binary probability model, such as logit or probit, $\beta_{1}$, $\beta_{2}$, and Γ do not represent the marginal effects. Instead, marginal effects are calculated as the function of the probability density and then translated into elasticities, typically at the level of average prices, income, and all the variables in the model.

#### 3.2.2. The Estimation of the Model and the Elasticities

The estimation of the model has several specificities. Firstly, since HBS data do not contain information on the prices of cigarettes, unit values are used as a proxy for cigarettes prices. The unit values are calculated as the ratio between total household expenditure on cigarettes (in local currency) and total household consumption of cigarettes (in cigarette packs). The potential identification problem due to joint determination of cigarette demand and prices is resolved by calculating prices as cluster averages. We defined clusters based on the information on municipalities and years, i.e., the cluster was defined as a municipality x in the year t. According to this definition, we generated 1823 clusters, which, on average, included about 34 households. The assumption behind our estimation strategy was that the cluster averages represent the market price, determined by cluster-level factors such as transport and production costs and taxes, as they do not depend on income and household characteristics, which vary within clusters [22]. Additionally, numerous previous studies have indicated that cigarettes prices can be treated as exogenous [4,23] even if coming from the same level of aggregation [24]. Finally, a considerable part of cigarettes prices consists of value added tax and excises, which are regulated by the state and not by demand for cigarettes. Since the prices were calculated at the cluster level, cluster standard errors were included in the specification of the model. Additionally, total household consumption was used as a proxy for household disposable income, as information on income was not consistently available in all the years.

Secondly, while the first part of the model was estimated via a simple logit function, the estimation of the second part of the model relied on Deaton’s demand model [9,22]. Deaton’s model is a consumer behavior model, which is preferred to simple OLS or GLM, as it provides a built-in identification strategy and controls for so-called quality shading and measurement error. Deaton’s model includes a three-stage estimation procedure, which is explained in more detail in Appendix B or Deaton’s original work [9,22]. Thirdly, although the literature suggests that two parts of the model (prevalence and intensity) can be estimated independently, total elasticity cannot be calculated as a simple sum of the two elasticities. Instead, this sum needed to be corrected for the fact that a change in the smoking prevalence can attenuate or enlarge the effect of the conditional demand (intensity) elasticity. A more detailed explanation of the calculation of the total elasticity can be found in Appendix C.

Finally, the focus of this research was to analyze the price elasticity of demand by income groups. Income groups were constructed based on total household consumption (a proxy for income) per capita. Three income groups were created: low-income, middle-income, and high-income. Since 12 waves of HBS were used, the division into three income groups was done for each year, so that an equal (weighted) number of households belonged to each of the three groups in all years. After dividing the sample into three subsamples according to income groups, prevalence elasticity and conditional demand (intensity) elasticity were estimated for each of the subsamples, using the same methods explained above.

## 4. Results

### 4.1. Estimated Elasticities

#### 4.1.1. Overall Elasticity

According to the estimates from the logit model, the price elasticity of smoking prevalence in Serbia amounted to −0.265 (Table 2). In other words, a 10% increase in the price of cigarettes decreases the probability of smoking at the household level by 2.65%. In absolute terms, a 10% increase in price would reduce the current prevalence by 0.9 percentage points, i.e., current prevalence would decrease from 34.2% to 33.3%. Semi-elasticities are presented in Table A1 in the Appendix. All other things being equal, households with higher income (that is, higher total expenditure) have higher levels of smoking prevalence, with an income elasticity of 0.609. In other words, a 10% higher income results in about 6% higher probability of smoking at the household level. The impact of other variables in the model was also in the line with expectations (Table A1 in Appendix A): Prevalence was higher in larger households and households with higher shares of men and adults. Education, conditional on all other variables, had a non-linear impact: The lowest prevalence was associated with the lowest (incomplete primary) and highest (tertiary) levels of education. Compared to Belgrade, all other regions had a higher prevalence. Pensioner and self-employed households had lower, while unemployed households had a higher prevalence than employed households. Finally, the introduction of the advertisement ban in 2010 has reduced smoking prevalence

Results robustness is confirmed by using a model in which price and income enter the model in linear rather than log form. Elasticities obtained were very similar to the ones from the log model. Initial estimates indicated that the square term for prices was insignificant and it was, therefore, omitted from the specification in Table A1.

On the other hand, results from the Deaton model indicated a negative intensity price elasticity of −0.395, i.e., if cigarette prices in Serbia increased by 10%, the number of cigarettes consumed by those who smoke would decrease by about 4%. The estimated value of conditional income elasticity was positive at 0.447. Robustness of the Deaton’s model was checked with the estimation within the GLM model. Results suggested similar price and income elasticities (Table A3 in the Appendix A). Table A2 presents the results of the first-stage Deaton equations, which were also in line with expectations. The coefficient for total expenditures in the unit value equation was significant, indicating that the quality shading effect cannot be neglected and a need for the application of Deaton’s model to obtain an unbiased estimate of the conditional demand elasticity. The remaining coefficients from unit value and budget share regressions also had the expected signs.

Based on the estimates of prevalence and conditional demand elasticities, total demand elasticity was calculated. As explained in the methodology section and Appendix C, total elasticity was calculated as a corrected, rather than a simple, sum of the two elasticities. More precisely, the size of the conditional demand elasticity needed to be corrected for the change in the number of smokers that occurs due to the increase/decrease in the prevalence. Total price elasticity amounted to −0.659, an estimate similar to previous estimates for Serbia [7].

#### 4.1.2. Elasticities by Income Groups

Table 2 also presents the elasticities by income group. In line with the previous research, the price elasticity of smoking prevalence was the highest for low-income households, estimated at −0.565. For the middle-income group, the elasticity was −0.216, while for high-income households, it was not significant, suggesting that, for the last group, the decision to smoke was not impacted by price. Price intensity elasticity was negative for all three groups and also the highest for low-income households, at −0.514. For middle- and high-income households, elasticities were also negative and estimated at −0.371 and −0.220, respectively. Similar results were obtained when the GLM method was applied to calculate elasticities (Table A6 in the Appendix A), thus confirming the robustness of the results. The analysis further indicated that, in all income groups, higher income increases smoking prevalence: Income elasticity was the highest for low-income households, at 0.809; slightly lower in the middle-income group, at 0.665; and the lowest in the high-income group, at 0.401. Income elasticity of conditional demand (intensity elasticity of income) was positive and estimated at 0.550, 0.598, and 0.338, for low-, middle-, and high-income households, respectively.

Based on the estimates of prevalence and conditional demand elasticity, total demand elasticity was calculated. Total price elasticity was the highest for low-income households, at −1.076, which means that a 10% price increase leads to a decrease in consumption by 10.8%. In the middle-income households, total elasticity was about two times lower, at −0.631, while the total price elasticity for the high-income group was −0.220 and was entirely attributable to a decrease in smoking intensity, as prices had no significant effect on prevalence. Total income elasticities in all the groups were higher than the total price elasticities and estimated at 1.363, 1.267, and 0.740 for low-, middle-, and high-income households, respectively.

### 4.2. Impact of Price Increases on Consumption and Tobacco Taxes

The obtained elasticities were used to simulate the effect that a price increase would have on cigarette consumption, government revenue from taxes on cigarettes, including both excises and value-added tax (VAT), and expenditures on cigarettes for the three groups. The results of the simulation are presented in Table 3. Total cigarette consumption in Serbia in 2017 was 671.4 million packs. The estimated total government revenue from cigarette consumption in 2017 was about 980 million euros (or 6.9% of the total government tax revenues). To obtain consumption by income groups, the total consumption of cigarettes was split by income group by applying the shares of total consumption calculated from HBS 2017 data (Column 1).

According to the Ministry of Finance, Tobacco Administration Department, the weighted average price of cigarettes in 2017 (the last year of the available data) was €1.87 (that is, 226.96 RSD). According to the taxation rules, a specific excise was set at €0.53 per pack (64.75 RSD), ad valorem excise at €0.62 per pack (33% of the retail price), and VAT at €0.31 per pack (20% of the pre-VAT price). Therefore, the total tax paid on a pack of cigarettes in Serbia amounted to €1.46 and it represents about 78.8% of the total retail price.

The effects on tobacco consumption (Column 2) were calculated as follows:

*D_t + 1_* = *D_t_* ∗ (1 + *E_p_* ∗ *%p change* + *E_i_* ∗ *%i change*) (3)

where *D_t + 1_* is the new demand, *D_t_* is the demand in year *t*, and *E_p_* and *E_i_* are price and income elasticities (by income groups, from Table 2), while *%p* and *%i* change are the percentage increases in price and income, respectively. An increase in income (*%i*) is based on the 2018 personal consumption growth rate of 3.0%, adjusted for the real growth in private consumption by income group, between 2016 and 2017, obtained from HBS data. For the low-, middle-, and high-income groups, estimated growth rates were 3.9, 3.1, and 2.0%, respectively. Assuming a 25% price increase (*%p)*, resulting from a 44% increase of specific excise, and no change in the net-of-tax price, the price would increase from €1.87 to €2.34 (full calculation is presented in Table A7 in Appendix A).

Due to the highest price elasticity, the low-income group would experience the largest reduction in consumption, at 21.6%, while the decrease for the middle- and high-income groups would be 11.8 and 4.0%, respectively (column 3).

The total spending on cigarettes for the three income groups for 2017 and 2018 (columns 4 and 5) was calculated as a product of consumption from 2017 and 2018 (columns 1 and 2) and the weighted average price of cigarettes €1.87 for 2017 and simulated price for 2018 that would include a 25% increase (€1.87 ∗ 1.25 = €2.34). The results suggested that low-income households (as a group) would decrease their total expenditures on cigarettes by about 2% (Column 6), as the overall decrease in their consumption was faster than the increase in prices (the demand is elastic, with elasticity higher than 1). On the other hand, middle- and high-income households would increase their expenditures by about 10% and 20%, respectively, as their demand for cigarettes was less elastic (Column 6).

Finally, the total government tax on tobacco that each group of households would pay in 2017 and 2018 (columns 7 and 8) was calculated as a product of consumption from 2017 and 2018 (columns 1 and 2) and total taxes paid on tobacco in 2017 (€1.46) and 2018 (€1.93, resulting from an increase in specific excise by 44% and adjustments of ad valorem excise and VAT; see Table A7 in Appendix A). The results (column 9) suggested that, while all the groups would pay higher tobacco taxes, the increase would be the lowest for low-income households (by 3.5%) and the highest for high-income households (26.7%). This means that, after the tax increase, low-income households would pay a lower share of overall taxes. In the baseline scenario, in 2017, low-income households paid about 24% of total tobacco taxes (236.3 out of 979.3 million euros, column 7), while, after the tax increase, they would pay about 21% (244.7 out of 1.150.5 million euros, column 8). At the same time, high-income households paid about 40% of total tobacco taxes in the baseline scenario (395.3 out of 979.3 million euros, column 7), and, after the tax increase, high-income households would pay about 43% (501 out of 1.150.5 million euros, column 8). In total, it was estimated that an increase of prices by 25% (resulting from a 44% increase in specific excise) would result in an increase of the government revenue from tobacco by 17.4%.

## 5. Conclusions

Previous research on tobacco elasticities for Serbia indicated that increasing tobacco excises is a crucial instrument for lowering the demand for tobacco products, which leads to lower health risks and higher levels of well-being. This policy has been criticized for its alleged regressive impact on the poor; however, this critique has neglected the decrease in consumption that occurs after tobacco excises and prices increase. The aim of this paper was to provide empirical arguments on the debate around the progressivity and inequality effects of tobacco tax increase while accounting for the change in the consumption patterns that would occur after the tax increase. In order to estimate the impact of increase in cigarette prices on demand for cigarettes, i.e., price elasticity of demand, we combined methods of two-part [8] and Deaton’s model [9] and used HBS data (from 2006 to 2017). Estimated elasticities were then used to calculate the effect on tobacco expenditures and taxes paid by the three groups.

The evidence presented in this paper is the first empirical evidence on the progressivity/regressivity of tobacco taxation in Serbia (and also in the wider Western Balkans region), when consumers’ responses are taken into account. Our results suggest that the excise increase would result in several favorable trends for low-income households. Firstly, we found that increasing excises, via decreasing demand, lowers tobacco expenditures for low-income households. For them, the resources used for cigarettes could then be used for more productive consumption, such as on food, clothes, etc. [25] This means that, after the tobacco tax increase, these households will be less likely to fall into secondary poverty—the situation in which the low-income household has enough resources but, due to inefficient use (such as on cigarettes), it has the same level of productive consumption as poor households. Therefore, by lowering cigarettes use after the excise increase, low-income households decrease the differences between them and other income groups in productive consumption.

Secondly, our analysis shows that the increase of tobacco taxes would result in a shift in the tax burden from low- towards high-income households. After a 25% price increase (resulting from a 44.1% increase of the specific excise), the share of total taxes paid by low-income households would decrease by about 3 percentage points (from 24 to 21%), while the share paid by high-income households would increase by approximately the same amount. This suggests that the increase of the tobacco taxes, due to a decrease in consumption, makes the distribution of tobacco taxes more progressive, as a higher share of the taxes would be paid by high-income households.

Thirdly, estimated elasticities suggest that, after a tax increase, all income groups decrease their tobacco consumption and, therefore, in the medium and long term improve their health and lower their medical expenditures. This decrease is most pronounced for the low-income households, which is important as persons from these households are most susceptible to tobacco-related illnesses and mortality [10]. Therefore, increasing excises will improve low-income households’ health outcomes the most and lower long-term health inequalities. Furthermore, in the long run, low-income households will lower medical expenditures the most therefore reducing future consumption inequalities.

Our research offers several other contributions to the existing literature on tobacco taxation and its effects, which have significant policy implications. Firstly, our research provides that results show that both prevalence and intensity of tobacco consumption are reduced with the tobacco price increase. This result is important as it shows that increasing tobacco prices can be used as an instrument to decrease both prevalence and intensity of smoking. Secondly, we provided estimates of the cigarettes price elasticities for different income groups, which is the first estimation of this kind for Serbia and, in general, in the region of the Western Balkans. The results suggest that the low-income households have the highest elasticity, and that a decrease of their demand is faster than the increase of prices. This is in line with previous research for high-, and low-, and middle-income countries, which also shows that the poorest have the highest responsiveness to the price increase. Finally, according to our estimations (Table 3), we showed that the specific excise increase of 44% would result in the overall tobacco tax revenues being higher by 17.4%, bringing an additional 170 million EUR in fiscal revenues. Although tobacco excises make about 5.4% of total tax revenue in Serbia [7], it is important to note that the total collected revenue would increase, therefore adding positive fiscal effects to the list of other positive consequences of the tobacco excise increase.

To summarize, this paper provides arguments that increasing tobacco excises has several positive effects. Firstly, we refuted the hypothesis that increasing tobacco taxes is regressive and showed that there are several positive outcomes for low-income households from the perspective of inequality. According to our results, a tobacco tax increase will result in lower inequalities in productive consumption, a shift in the tobacco tax burden from low- towards high-income households, and lower long-term health and consumption inequalities. Secondly, we provided strong evidence that an excise increase would result in a reduction in both prevalence and intensity of cigarettes use for all income groups, which lowers the health risks of tobacco use. The decrease will be the highest for low-income households, who are at a greater risk of tobacco-related diseases and mortality. Finally, increasing excises will, due to the fact that average price elasticity is lower than 1 (i.e., inelastic demand), lead to an increase in fiscal revenues.

## Figures and Tables

**Table 1:** Cigarette use in Serbia: prevalence, expenditures, number of consumed cigarettes.

Year	Smoking Prevalence (% of Households)	The Average Number of Cigarettes Smoked (Packs Per Household) ^1^	Average Real Household Expenditure on Cigarettes (in RSD) ^1,2^	Average Real Price (in RSD) ^1,2,3^
2006	49.7	39.1	1988	51.9
2007	47.9	39.2	2279	58.7
2008	44.1	39.0	2268	58.9
2009	42.0	37.9	2353	62.7
2010	38.8	37.0	2442	65.9
2011	38.4	36.2	2487	68.7
2012	38.0	34.3	2609	75.8
2013	35.1	29.6	2758	93.0
2014	34.4	27.7	2922	104.9
2015	36.3	28.9	2985	103.2
2016	33.7	29.1	3219	110.2
2017	34.2	27.2	3241	117.8

Source: Authors’ calculation based on HBS data for Serbia. ^1^ Based on reported expenditure and quantities of households with positive expenditure on cigarettes. ^2^ Variables deflated by CPI to 2006 values. ^3^ The average price was proxied by the average unit value, which is a ratio of reported household expenditure on cigarettes and purchased quantity.

**Table 2:** Prevalence and conditional demand elasticities by income group.

	All Households ^1^		Low-Income Households ^2^		Middle-Income Households ^2^		High-Income Households ^2^	
Prevalence elasticities (logit model) ^3^								
Price	−0.265 ***	(0.050)	−0.565 ***	(0.075)	−0.261 ***	(0.070)	−0.040	(0.066)
Income	0.609 ***	(0.020)	0.809 ***	(0.044)	0.665 ***	(0.062)	0.401 ***	(0.031)
Conditional demand (intensity) elasticity (Deaton’s model)								
Price	−0.395 ***	(0.053)	−0.514 ***	(0.067)	−0.371 ***	(0.065)	−0.220 ***	(0.041)
Income	0.447 ***	(0.011)	0.550 ***	(0.037)	0.598 ***	(0.065)	0.338 ***	(0.025)
Total elasticity ^4^								
Price	−0.659		−1.076		−0.631		−0.220	
Income	1.058		1.363		1.267		0.740	

Source: Authors’ calculation based on HBS data Significance levels: *** *p* < 0.01, ** *p* < 0.05, * *p* < 0.1. Notes: ^1^ Full estimates of the cigarettes prevalence model are presented in Table A1 (column log model) in Appendix A. First-stage equations of the Deaton model for all households and Table A2 in Appendix A. ^2^ Estimates of the cigarettes prevalence model and first-stage equations of the Deaton model for three income groups are presented in Table A4 and Table A5 (Appendix A), respectively. ^3^ Estimated elasticities represent marginal effects calculated based on the logit model (Table A1 and Table A4), at the level of average values of all other variables included in the model. ^4^ Total elasticity is a corrected, rather than a simple, sum of the two elasticities. The size of the conditional demand elasticity was corrected for the change in the number of smokers that occurs due to the increase/decrease in the prevalence. Due to the way of calculating the overall elasticity the significance levels were not calculated (see Section 3.2).

**Table 3:** Impact of price increase on consumption and expenditures by income group.

Income Group	Consumption			Spending on Cigarettes			Government Revenue		
	2017 ^1^	2018 ^1,^*	Change	2017 ^2^	2018 ^2^^,^*	Change	2017 ^2^	2018 ^2^^,^*	Change
	1	2	3	4	5	6	7	8	9
Low	162.0	127.0	−21.6%	302.9	296.9	−2.0%	236.3	244.7	3.5%
Middle	238.4	210.1	−11.8%	445.8	491.2	10.2%	347.7	404.8	16.4%
High	271.0	260.1	−4.0%	506.8	608.0	20.0%	395.3	501.0	26.7%
Total	671.4	597.3	−11.0%	1255.4	1396.1	11.2%	979.3	1150.5	17.4%

Notes: ^1^ In million packs; ^2^ in million euros * Simulated values. Source: Authors’ calculation based on HBS, Ministry of Finance data, and estimated elasticities.

## Data Availability

The data presented in this study are available on request from the corresponding author.

## Appendix A

**Table A1:** Smoking prevalence model (different specifications).

VARIABLES	Linear Price and Income		Log Price and Income	
Price	−0.005 ***	(0.001)	−0.436 ***	(0.083)
Income	0.544 ***	(0.020)	1.360 ***	(0.058)
Income squared	−0.033 ***	(0.002)	−0.215 ***	(0.023)
Household size	0.075 ***	(0.009)	0.072 ***	(0.009)
Male ratio	0.494 ***	(0.037)	0.490 ***	(0.038)
Adult ratio	0.490 ***	(0.064)	0.488 ***	(0.064)
Education: Incomplete primary				
Primary	0.630 ***	(0.048)	0.524 ***	(0.050)
Secondary (2–3 years)	0.741 ***	(0.049)	0.585 ***	(0.051)
Secondary (4 years)	0.462 ***	(0.050)	0.296 ***	(0.051)
Tertiary (2 years)	0.208 ***	(0.056)	0.047	(0.057)
Tertiary (3+ years)	−0.079	(0.055)	−0.225 ***	(0.056)
Region: Belgrade				
Vojvodina	0.132 ***	(0.051)	0.144 ***	(0.051)
West	0.244 ***	(0.050)	0.240 ***	(0.050)
East	0.095 *	(0.055)	0.102 *	(0.055)
HH Activity: Employed				
Unemployed	0.043	(0.049)	0.156 ***	(0.051)
Pensioners	−0.629 ***	(0.027)	−0.618 ***	(0.027)
Self-employed	−0.184 ***	(0.030)	−0.195 ***	(0.030)
Advertisement ban (2010–2017 = 1)	−0.218 ***	(0.050)	−0.202 ***	(0.053)
Constant	−2.384 ***	(0.120)	−0.554	(0.358)
Marginal effects (elasticities)				
Price	−0.269 ***	(0.051)	−0.265 ***	(0.050)
Income	0.467 ***	(0.016)	0.609 ***	(0.020)
Marginal effects (perc. points) ^1^				
Price	−0.089 ***	(0.017)	−0.090 ***	(0.017)
Income	0.167 ***	(0.005)	0.181 ***	(0.005)
BIC	73,973.5		73967.9	
Pseudo R square	0.113		0.113	
Log likelihood	−36,876.4		−36,879.1	
Observations	62,051		62,051	

Notes: ^1^ Unit change of prevalence (in percentage points), resulting from a percentage change in prices. Cluster robust standard errors in parentheses; *** *p* < 0.01, ** *p* < 0.05, * *p* < 0.1.

**Table A2:** First-stage regression results for Deaton model.

VARIABLES	Unit Value (Per Pack, ln)		Cigarettes’ Budget Share (in %)	
Total expenditure (ln)	0.114 ***	(0.003)	−0.034 ***	(0.001)
Household size (ln)	−0.060 ***	(0.003)	−0.003 ***	(0.001)
Male ratio	−0.026 ***	(0.005)	0.019 ***	(0.001)
Adult ratio	−0.040 ***	(0.008)	0.009 ***	(0.002)
Mean education	−0.007 ***	(0.001)	−0.001**	(0.000)
Maximum education	0.001	(0.001)	−0.001 ***	(0.000)
Rural Settlements	−0.023 ***	(0.003)	0.000	(0.001)
Household type—Employed	omitted			
Unemployed	0.014 **	(0.006)	0.007 ***	(0.002)
Pensioners	−0.030 ***	(0.003)	−0.006 ***	(0.001)
Self-employed	0.002	(0.004)	−0.001	(0.001)
Cluster dummies	F(1671, 22,461) = 42.513 ***		F(736, 9243) = 2.954 ***	
Constant	3.198 ***	(0.030)	0.440 ***	(0.008)
Observations	24,143		24,143	
R-squared	0.780		0.320	

Standard errors in parentheses, *** *p* < 0.01, ** *p* < 0.05, * *p* < 0.1. Authors’ calculation based on the HBS data.

**Table A3:** Conditional demand model (GLM estimate).

VARIABLES	Linear Price and Income		Log Price and Income	
Price	−0.005 ***	(0.000)	−0.450 ***	(0.031)
Income	0.217 ***	(0.008)	0.558 ***	(0.024)
Income squared	−0.011 ***	(0.001)	−0.059 ***	(0.009)
Household size	0.039 ***	(0.003)	0.039 ***	(0.003)
Male ratio	0.186 ***	(0.016)	0.194 ***	(0.016)
Adult ratio	0.275 ***	(0.026)	0.282 ***	(0.026)
Education: Incomplete primary				
Primary	0.039	(0.027)	−0.001	(0.027)
Secondary (2–3 years)	0.024	(0.026)	−0.031	(0.026)
Secondary (4 years)	−0.070 ***	(0.027)	−0.127 ***	(0.026)
Tertiary (2 years)	−0.113 ***	(0.029)	−0.169 ***	(0.029)
Tertiary (3+ years)	−0.182 ***	(0.030)	−0.237 ***	(0.029)
Region: Belgrade				
Vojvodina	0.037 *	(0.019)	0.030	(0.020)
West	0.073 ***	(0.019)	0.064 ***	(0.019)
East	0.145 ***	(0.019)	0.134 ***	(0.019)
HH Activity: Employed				
Unemployed	0.026	(0.020)	0.061 ***	(0.020)
Pensioners	−0.045 ***	(0.012)	−0.038 ***	(0.012)
Self-employed	−0.027 **	(0.012)	−0.027 **	(0.012)
Advertisement ban (2010–2017 = 1)	−0.025	(0.017)	−0.011	(0.017)
Constant	2.853 ***	(0.049)	4.453 ***	(0.132)
Marginal effects (elasticities)				
Price	−0.446 ***	(0.031)	−0.450 ***	(0.031)
Income	0.391 ***	(0.011)	0.413 ***	(0.012)
BIC	218,705		218,647	
Log likelihood	−109,257		−109,227	
Observations	24,356		24,356	

Cluster robust standard errors in parentheses, *** *p* < 0.01, ** *p* < 0.05, * *p* < 0.1.

**Table A4:** Estimation of the prevalence elasticity by income groups.

VARIABLES	Low Income		Mid Income		High Income	
Price (ln)	−0.873 ***	(0.116)	−0.433 ***	(0.116)	−0.069	(0.115)
Income (ln)	1.311 ***	(0.086)	1.417 ***	(0.115)	1.229 ***	(0.126)
Income (ln) squared	−0.062	(0.050)	−0.185 ***	(0.069)	−0.219 ***	(0.041)
Household size	0.068 ***	(0.018)	0.051	(0.038)	0.075 ***	(0.021)
Male ratio	0.686 ***	(0.081)	0.706 ***	(0.070)	0.279 ***	(0.051)
Adult ratio	0.283 ***	(0.100)	0.768 ***	(0.110)	0.464 ***	(0.133)
Education: Incomplete primary	(ommited)					
Primary	0.369 ***	(0.081)	0.585 ***	(0.081)	0.625 ***	(0.091)
Secondary (2–3 years)	0.371 ***	(0.082)	0.633 ***	(0.082)	0.830 ***	(0.086)
Secondary (4 years)	0.053	(0.083)	0.353 ***	(0.080)	0.557 ***	(0.086)
Tertiary (2 years)	−0.251**	(0.098)	0.033	(0.093)	0.375 ***	(0.091)
Tertiary (3+ years)	−0.483 ***	(0.098)	−0.159*	(0.090)	−0.007	(0.093)
Region: Belgrade	(ommited)					
Vojvodina	0.160 *	(0.086)	0.271 ***	(0.075)	0.033	(0.059)
West	0.083	(0.082)	0.362 ***	(0.074)	0.277 ***	(0.058)
East	−0.090	(0.089)	0.263 ***	(0.080)	0.197 ***	(0.064)
HH Activity: Employed	(ommited)					
Unemployed	0.294 ***	(0.071)	0.122	(0.100)	−0.006	(0.103)
Pensioners	−0.402 ***	(0.049)	−0.641 ***	(0.050)	−0.753 ***	(0.041)
Self-employed	−0.089 *	(0.049)	−0.212 ***	(0.052)	−0.272 ***	(0.051)
Advertisement ban (2010–2017 = 1)	−0.134 *	(0.076)	−0.214 ***	(0.077)	−0.253 ***	(0.072)
Constant	1.530 ***	(0.492)	−1.119 **	(0.515)	−1.975 ***	(0.502)
Marginal effects (%, elasticities)						
Price	−0.565 ***	(0.075)	−0.261 ***	(0.070)	−0.040	(0.066)
Income	0.809 ***	(0.044)	0.665 ***	(0.062)	0.401 ***	(0.031)
Marginal effects (percentage points)						
Price	−0.163 ***	(0.022)	−0.087 ***	(0.024)	−0.018	(0.025)
Income	0.232 ***	(0.013)	0.204 ***	(0.025)	0.135 ***	(0.011)
Observations	20,687		20,684		20,680	

Cluster robust standard errors in parentheses, *** *p* < 0.01, ** *p* < 0.05, * *p* < 0.1.

**Table A5:** Unit value and budget share equations from Deaton model by income groups.

	Low-Income Hh		Mid-Income hh		High-Income Hhs	
VARIABLES	Unit Value	Budget Share	Unit Value	Budget Share	Unit Value	Budget Share
Total expenditure (ln)	0.104 ***	−0.031 ***	0.112 ***	−0.023 ***	0.110 ***	−0.037 ***
	(0.008)	(0.003)	(0.017)	(0.005)	(0.008)	(0.002)
Household size (ln)	−0.057 ***	−0.007 **	−0.052 ***	−0.015 ***	−0.060 ***	0.001
	(0.009)	(0.003)	(0.018)	(0.005)	(0.009)	(0.002)
Male ratio	−0.020 *	0.021 ***	−0.024 **	0.020 ***	−0.027 ***	0.017 ***
	(0.010)	(0.004)	(0.010)	(0.003)	(0.008)	(0.002)
Adult ratio	−0.019	0.007	−0.048 ***	0.005	−0.068 ***	0.014 ***
	(0.012)	(0.004)	(0.014)	(0.004)	(0.018)	(0.004)
Mean education	0.005 ***	−0.002 ***	0.002	−0.001*	0.013 ***	0.001
	(0.002)	(0.001)	(0.002)	(0.001)	(0.002)	(0.001)
Maximum education	0.001	−0.000	0.003	−0.001**	−0.002	−0.001 ***
	(0.002)	(0.001)	(0.002)	(0.000)	(0.002)	(0.000)
Rural Settlements	0.014 ***	−0.001	0.026 ***	0.002	0.025 ***	0.000
	(0.005)	(0.002)	(0.005)	(0.002)	(0.006)	(0.001)
Household type—Employed	Omitted		Omitted		Omitted	
Unemployed	0.013	0.009 ***	0.002	0.012 ***	0.019	−0.002
	(0.009)	(0.003)	(0.013)	(0.004)	(0.014)	(0.003)
Pensioners	−0.012 *	−0.005 **	−0.025 ***	−0.003*	−0.044 ***	−0.010 ***
	(0.006)	(0.002)	(0.006)	(0.002)	(0.006)	(0.001)
Self-employed	−0.001	−0.002	−0.003	−0.001	0.009	−0.001
	(0.006)	(0.002)	(0.007)	(0.002)	(0.008)	(0.002)
Constant	4.199 ***	0.136 ***	4.250 ***	0.131 ***	4.234 ***	0.111 ***
	(0.020)	(0.007)	(0.021)	(0.006)	(0.024)	(0.005)
Number of clusters	1476	1476	1514	1514	1384	1384
Cluster effect F =	22.101 ***	1.625 ***	16.471 ***	1.783 ***	13.988 ***	1.993 ***
Observations	7265	7265	8186	8186	8692	8692
R-squared	0.860	0.373	0.810	0.404	0.743	0.417

**Notes:** *** *p* < 0.01, ** *p* < 0.05, * *p* < 0.1.

**Table A6:** GLM estimation of the intensity elasticity by income groups.

VARIABLES	Low-Income		Mid-Income		High-Income	
Price	−0.605 ***	(0.044)	−0.441 ***	(0.042)	−0.348 ***	(0.043)
Income	0.562 ***	(0.037)	0.472 ***	(0.051)	0.514 ***	(0.055)
Income squared	0.003	(0.021)	0.002	(0.029)	−0.073 ***	(0.018)
Household size	0.024 ***	(0.007)	0.019	(0.015)	0.056 ***	(0.009)
Male ratio	0.194 ***	(0.036)	0.165 ***	(0.031)	0.204 ***	(0.022)
Adult ratio	0.178 ***	(0.041)	0.271 ***	(0.042)	0.414 ***	(0.053)
Education: Incomplete primary						
Primary	−0.032	(0.040)	0.035	(0.040)	0.007	(0.057)
Secondary (2–3 years)	−0.092 **	(0.040)	0.021	(0.039)	0.025	(0.055)
Secondary (4 years)	−0.179 ***	(0.041)	−0.098 **	(0.039)	−0.063	(0.055)
Tertiary (2 years)	−0.205 ***	(0.049)	−0.144 ***	(0.043)	−0.111 *	(0.059)
Tertiary (3+ years)	−0.312 ***	(0.050)	−0.196 ***	(0.043)	−0.176 ***	(0.058)
Region: Belgrade						
Vojvodina	−0.059 *	(0.032)	0.036	(0.027)	0.063 ***	(0.024)
West	0.027	(0.031)	0.054 **	(0.026)	0.078 ***	(0.024)
East	0.084 ***	(0.032)	0.139 ***	(0.027)	0.148 ***	(0.025)
HH Activity: Employed						
Unemployed	0.118 ***	(0.031)	0.096 **	(0.038)	−0.055	(0.038)
Pensioners	0.010	(0.022)	−0.020	(0.021)	−0.098 ***	(0.020)
Self-employed	−0.006	(0.020)	−0.029	(0.019)	−0.038 *	(0.021)
Advertisement ban (2010–2017 = 1)	−0.025	(0.027)	0.003	(0.026)	−0.016	(0.026)
Constant	5.284 ***	(0.195)	4.462 ***	(0.186)	3.881 ***	(0.194)
Marginal effects (elasticities)						
Price	−0.605 ***	(0.044)	−0.441 ***	(0.042)	−0.348 ***	(0.043)
Income	0.562 ***	(0.037)	0.472 ***	(0.051)	0.514 ***	(0.055)
Observations	7309		8220		8827	

**Notes:** *** *p* < 0.01, ** *p* < 0.05, * *p* < 0.1.

**Table A7:** Calculation of new price for 2018 by price components.

	2017	2018	Change
Retail Price (Weighted average price—WAP)	1.87	2.34	25%
Excise (specific)	0.53	0.77	44.1%
Excise (ad valorem, 33% of WAP)	0.62	0.77	25.0%
Total excise	1.15	1.54	33.9%
VAT	0.31	0.39	25.0%
Total Tax	1.46	1.93	32.0%
Net-of-tax price	0.41	0.41	0.0%

## Appendix B

The starting point of Deaton’s model are two equations:

(A1) $$w_{hc}=\alpha^{0}+\beta^{0}lnx_{hc}+\gamma^{0}\cdot z_{hc}+\theta lnp_{c}+\left(f_{c}+u_{ch}^{0}\right)$$

(A2) $$lnv_{hc}=\alpha^{1}+\beta^{1}lnx_{hc}+\gamma^{1}\cdot z_{hc}+\psi lnp_{c}+u_{hc}^{1}$$

where indices *h* and *c* represent households and clusters, respectively. The left-hand-side variables in Equations (A1) and (A2) are $w_{hc}$, share of the household budget spent on cigarettes (in percentages), and the natural logarithm of $v_{hc}$, cigarette unit values. On the right-hand side of both equations, $x_{hc}$ represents total expenditures of the household *h* in cluster *c*, $z_{hc}$ is other household characteristics, $p_{c}$ is the price of the cigarettes in cluster c, while $u_{ch}^{0}$ and $u_{hc}^{1}$ represent the error term. Finally, in Equation (A1) $f_{c}$ is the cluster level effects on the budget share, which are assumed to be uncorrelated with the price effect on the budget share. Since the prices were not observed, the parameters θ and ψ cannot be directly estimated from Equations (A1) and (A2). However, the assumption that market prices do not vary within the cluster (hence, the absence of the index *h* next to prices) enables consistent estimates of the remaining parameters. Cross-cluster variation in budget shares and unit values implicitly serves as an instrument for the identification of the demand function; the prices are exogenous, as they come from a higher level of aggregation.

In the second stage, estimates from the first stage (estimation of Equations (A1) and (A2)) were used to remove the effects of the total household expenditure and other household characteristics from the budget shares and the unit values. Residual variables were then used to create cluster averages of budget shares and unit values, which were then used as dependent variables

(A3) $$w_{c}^{\prime }=\alpha^{0}+\theta lnp_{c}+f_{c}+u_{c}^{0}$$

(A4) $${lnv}_{c}^{\prime }=\alpha^{1}+\psi lnp_{c}+u_{c}^{1}$$

The estimation of the parameter θ was still not possible, as the price was not observed. However, Deaton’s model uses the presence of price in both equations to establish a relationship between budget shares and unit values:

(A5) $$w_{c}^{\prime }=\alpha^{0}+\frac{\alpha^{1}}{\psi }+\theta \left(\frac{1}{\psi }{lnv}_{c}^{\prime }\right)+f_{c}+u_{c}^{0}+\frac{u_{c}^{1}}{\psi }$$

The result is the hybrid parameter of price and quality elasticity $\psi^{-1}\theta =\phi$, which can be estimated in the (A5). In the third stage, the weak separability assumption is introduced. Given that the budget share is defined as the product of the quantity of cigarettes and unit value divided by total expenditures, parameter θ can be estimated as:

(A6) $$\hat{\theta }=\hat{\phi }/\left[1+\left(w-\hat{\phi }\right)\frac{{\hat{\beta }}^{1}}{{\hat{\beta }}^{0}+w\left(1-{\hat{\beta }}^{1}\right)}\right]$$

where ${\hat{\beta }}^{1}$ and ${\hat{\beta }}^{0}$ are coefficients estimated in Equations (A1) and (A2), while *w* is the average value of the budget share. The value of $\hat{\psi }$ is then equal to ${\hat{\phi }}^{-1}\hat{\theta }$. From there, the price elasticity of demand can be estimated as:

(A7) $${\hat{\epsilon }}_{p}=\left(\frac{\hat{\theta }}{w}\right)-\hat{\psi }$$

## Appendix C

Although the literature [26] suggests that these two parts of the model, prevalence model and conditional demand (intensity) model, can be modeled independently, total elasticity cannot be calculated as a simple sum of the two elasticities. Instead, this sum needs to be corrected for the fact that a change in the smoking prevalence can attenuate or enlarge the effect of the conditional demand (intensity) elasticity. In order to make this clearer, an example is provided with the formula that converts the two elasticities into total elasticity.

Assume that the total population of a country is 10 million people, that that country has a prevalence rate of 40%, and that conditional average consumption per person is 25 cigarettes per day (including only those people who smoke). This means that about 4 million people smoke, and total consumption amounts to 100 million cigarettes per day. This situation is presented in Table A8 (column baseline).

**Table A8:** Hypothetical example for the calculation of the total demand elasticity.

		Baseline	Price Increases by 1%	% Change
Total population	1	10,000,000	10,000,000	
Prevalence	2	40.0%	39.88%	−0.30%
Consumption per person (in cigarettes)	3	25	24.875	−0.50%
Number of people smoking	4 = 1 × 2	4,000,000	3,988,000	−0.30%
Total consumption	5 = 4 × 3	100,000,000	99,201,500	−0.7985%

Also assume that the prevalence price elasticity in a country is −0.3, while the conditional demand (intensity) elasticity is −0.5. This means that if the prices increase by 1%, the prevalence would be lower by 0.3% (that is, to 39.88%), while the consumption per person would be lower by 0.5% (that is, to 24.875 cigarettes per day). This decreases the number of people smoking to 3.988 million (that is, by 0.3%), but the total consumption calculated as the product of new prevalence and consumption would decrease by −0.7985%, which is less than a simple sum of two elasticities of 0.8%. Therefore, due to the prevalence change, a total change in consumption will not be a simple sum of the two elasticities, so the change in prevalence should be corrected when adding up the change in consumption.

More formally, the total elasticity can be calculated according to the following formula:

(A8) $$\xi_{p}=\xi_{p1}+\left(1+\xi_{p1}\right)*\xi_{p2}$$

where $\xi_{p1}$ represents the prevalence elasticity, $\xi_{p2}$ represents the conditional demand (intensity) elasticity, and $\xi_{p}$ represents the total elasticity, if all the elasticities are expressed as percentages.
